# *Leishmania donovani* Infection with Atypical Cutaneous Manifestations, Himachal Pradesh, India, 2014–2018

**DOI:** 10.3201/eid2608.191761

**Published:** 2020-08

**Authors:** Lovlesh Thakur, Kiran K. Singh, Hemant R. Kushwaha, Sudarshan K. Sharma, Vinay Shankar, Ajeet Negi, Ghanshyam Verma, Sandhya Kumari, Aklank Jain, Manju Jain

**Affiliations:** Central University of Punjab, Bathinda, India (L. Thakur, K.K. Singh, A. Jain, M. Jain);; Jawaharlal Nehru University, New Delhi, India (H.R. Kushwaha);; Indira Gandhi Medical College, Shimla, India (S.K. Sharma, A. Negi, G. Verma, S. Kumari);; Maharishi Markandeshwar Medical College and Hospital, Kumarhatti-Solan, India (V. Shankar)

**Keywords:** atypical, cutaneous leishmaniasis, *Leishmania donovani*, parasites, Himachal Pradesh, India

## Abstract

We conducted a molecular study of parasite sequences from a cohort of cutaneous leishmaniasis patients in Himachal Pradesh, India. Results revealed atypical cutaneous disease caused by *Leishmania donovani* parasites. *L. donovani* variants causing cutaneous manifestations in this region are different from those causing visceral leishmaniasis in northeastern India.

Leishmaniasis is a complex disease with cutaneous, mucocutaneous, or visceral manifestations depending on the parasite species and host immunity. Despite continued elimination efforts, leishmaniasis continues to afflict known and newer endemic regions, where 0.5–0.9 million new cases of visceral leishmaniasis (VL) and 0.6–1.0 million new cases of cutaneous leishmaniasis (CL) occur every year (*1*). An increase in VL and CL cases from newer foci and atypical disease manifestation pose a challenge to leishmaniasis control programs (*2*–*7*). Unlike the known species-specific disease phenotype, parasite variants can cause atypical disease, so that *Leishmania* species generally associated with VL can cause CL and vice versa. 

In India, VL caused by *L. donovani* parasites in the northeastern region and CL caused by *L. tropica* in the western Thar Desert represent the prevalent forms of the disease (*2*). Himachal Pradesh is a more recently leishmaniasis-endemic state in northwest where VL and CL coexist; CL incidence is higher than VL incidence and most cases are attributable to *L. donovani* instead of *L. tropica* infection (*8*,*9*). Sharma et al. conducted limited molecular analysis of a few CL cases and reported preliminary findings (*8*). For an in-depth study on the involvement of *L. donovani* parasites in CL cases, we conducted a comprehensive molecular analysis of CL cases in Himachal Pradesh.

## The Study

**Figure 1:**
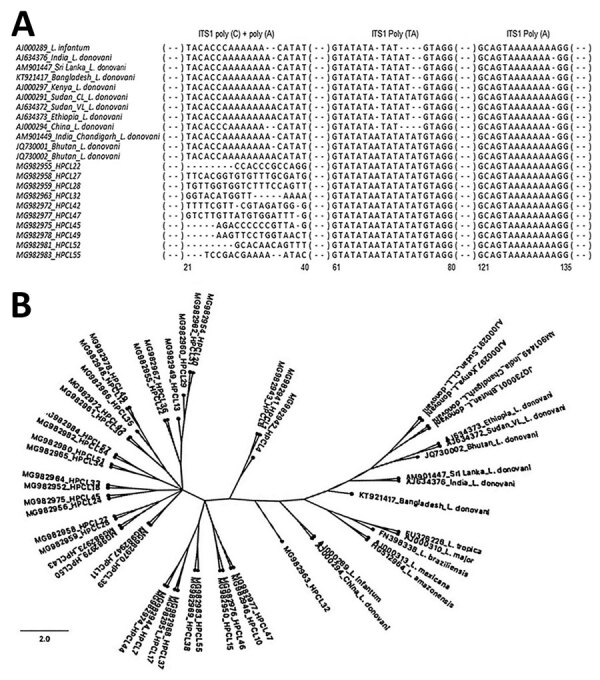
ITS1-based molecular analysis of clinical isolates from cutaneous leishmaniasis (CL) patients, Himachal Pradesh, India, 2014–2018. A) Multiple sequence alignment of ITS1 microsatellite repeat sequences of representative parasite isolates from CL patients with those of *L. donovani* complex reference strains from different geographic regions. Sequences were aligned by using BioEdit sequence alignment program (https://bioedit.software.informer.com/7.2). B) Phylogenetic tree of ITS1 sequences from CL test isolates (designated as HPCL, numbered in order of their collection) and standard *Leishmania* strains. Tree constructed by using maximum-likelihood method with 5,000 bootstraps in the *dnaml* program of PHYLIP package (http://evolution.genetics.washington.edu/phylip/doc/main.html). GenBank accession numbers are indicated. Scale bar indicates the nucleotide substitution per site. ITS1, internal transcribed spacer 1; RFLP, restriction fragment length polymorphism.

During 2014–2018, an increase in CL cases occurred in Himachal Pradesh; case reports came from different tehsils (i.e., townships) in Kinnaur, Shimla, and Kullu and the previously nonendemic districts of Mandi and Solan (Appendix Table 1, Figure 1). We confirmed 60 CL cases indigenous to the state with detailed patient information, demonstration of the presence of Leishman-Donovan bodies and CL-specific histopathologic changes in skin lesional specimens, and PCR detection of parasitic infection (Appendix).

We conducted PCR and restriction fragment-length polymorphism (RFLP) analysis of parasite species–specific internal transcribed spacer 1 (ITS1) sequences by using appropriate standard controls. We detected the expected ≈320-bp product with a *Hae*III RFLP pattern specific to *L. donovani* complex in all patient biopsy specimens, indicating *L. donovani*, *L. infantum,* or both as the causative agent of infection (Appendix Figure 4) (*10*). 

BLAST analysis (https://blast.ncbi.nlm.nih.gov/Blast.cgi) of 44 ITS1 test sequences showed all the samples to be closest to *L. donovani*, having maximum identity to *L. donovani* isolates from Bhutan (GenBank accession nos. JQ730001–2) and possibly *L. infantum*. None of the CL cases were consistent with *L. tropica* infection, unlike in a previous report (*8*). To distinguish whether HP isolates were *L. donovani*, *L. infantum*, or both and to infer genetic and geographic relatedness between these isolates and standard reference strains, we performed ITS1 microsatellite repeat analysis and phylogenetic classification (*11*–*13*). The 4 ITS1 polymorphic microsatellite repeat analysis indicate HP isolates different from *L. infantum* and closest to the *L. donovani* isolates from Bhutan (Table 1; Figure 1, panel A). We detected a polymorphism in the third poly (TA) microsatellite locus with 5 repeats and an atypical insert of TAA and the fourth poly (A) microsatellite tract with 8 repeats; these polymorphisms were identical to the VL-causing *L. donovani* isolates from Bhutan. An *L. donovani* Chandigarh isolate originally from HP is reported to be closest to the Bhutan isolates and matched with HP isolates at the third poly (TA) stretch (*12*). However, Himachal Pradesh isolates were distinct at the first poly C and the second poly A microsatellite tracts and had heterogeneous base sequences. Thus, these isolates represent *L. donovani* genetic variants; none showed the ITS1 sequence type previously assigned to the referred *L. donovani* isolates by Kuhls et al. (*13*). Our phylogenetic analysis of 44 ITS1 test sequences and ITS1 reference sequences placed all the CL-causing *L. donovani* isolates from Himachal Pradesh into a discrete cluster different from the VL-causing *L. donovani* from India and elsewhere and the CL-causing *L. donovani* isolates from Sri Lanka. The Himachal Pradesh CL isolates within the cluster exhibited considerable heterogeneity (Table 1; Figure 1, panel B; Appendix Table 4).

**Table 1:** Standard *Leishmania* strains used in ITS1-based microsatellite polymorphism and phylogenetic analysis of cutaneous leishmaniasis isolates, Himachal Pradesh, India, 2014–2018*

Standard *Leishmania* strains (place of origin)	WHO code	Genbank accession no.	Zymodeme	Disease form	Strain type†	ITS1 polymorphic microsatellite stretches (nucleotide position, bp)			
						Poly C (24–39)	Poly A (24–39)	Poly TA (61–76)	Poly A (124–134)
VL- and CL-causing *L. infantum* and *L. donovani* parasite strains									
* L. infantum* (Tunisia)	MHOM/TN/80/IPT1	AJ000289	MON-1	VL	A	3	6	4	8
* L. donovani* (India)	MHOM/IN/00/DEVI	AJ634376	MON-2	VL	H	2	8	5	7
* L. donovani* (Sri Lanka)	MHOM/LK/2002/ L60c	AM901447	MON-37	CL	ND	2	8	5	7
* L. donovani* (Bangladesh)	ND	KT921417	ND	VL	ND	2	8	5	7
* L. donovani* (Kenya)	MHOM/KE/85/ NLB323	AJ000297	MON-37	VL	G	2	8	5	7
* L. donovani* (Sudan)	MHOM/SD/75/ LV139	AJ000291	ND	CL	E	2	8	6	8
	MHOM/SD/93/9S	AJ634372	MON-18	VL	F	2	9	5	7
* L. donovani* (Ethiopia)	MHOM/ET/67/HU3	AJ634373	MON-18	VL	F	2	9	5	7
* L. donovani* (China)	MHOM/CN/00/ Wangjie1	AJ000294	MON-35	VL	C	3	6	4	7
* L. donovani* (HP, India)	MHOM/IN/83/ CHANDIGARH	AM901449	MON-37	VL	ND	2	8	2, TAA, 3	7
* L. donovani* (Bhutan)	Trashigang1	JQ730001	ND	VL	ND	2	8	2, TAA, 3	8
	Samtse1	JQ730002	ND	VL	ND	2	9	2, TAA, 3	8
CL-causing *L. donovani* isolates from Himachal Pradesh‡									
HPCL22	–	MG982955	ND	CL	ND	Heterogeneous		2, TAA, 3	8
HPCL27	–	MG982958	ND	CL	ND	Heterogeneous		2, TAA, 3	8
HPCL28	–	MG982959	ND	CL	ND	Heterogeneous		2, TAA, 3	8
HPCL32	–	MG982963	ND	CL	ND	Heterogeneous		2, TAA, 3	8
HPCL42	–	MG982972	ND	CL	ND	Heterogeneous		2, TAA, 3	8
HPCL45	–	MG982975	ND	CL	ND	Heterogeneous		2, TAA, 3	8
HPCL47	–	MG982977	ND	CL	ND	Heterogeneous		2, TAA, 3	8
HPCL49	–	MG982978	ND	CL	ND	Heterogeneous		2, TAA, 3	8
HPCL52	–	MG982981	ND	CL	ND	Heterogeneous		2, TAA, 3	8
HPCL55	–	MG982983	ND	CL	ND	Heterogeneous		2, TAA, 3	8
CL-causing standard WHO *Leishmania* species									
* L. major*	MHOM/SU/73/ 5ASKH	AJ000310	MON-4	CL	ND	4	6	6	6
* L. tropica*	MHOM/SU/60/OD	EU326226	LON-7	CL	ND	4	9	1, TTA, 2	3,C,4A
* L. mexicana*	MHOM/MX/85/ SOLIS	AJ000313	MON-152	CL	ND	2	8	1,	3,C,7A
* L. braziliensis*	MHOM/BR/00/ LTB300	FN398338	MON-166	CL	ND	2	6	1	5
* L. amazonensis*	MHOM/BR/73/ M2269	HG512964	MON-132	CL	ND	2	7	1	3,C,6A

*CL, cutaneous leishmaniasis; HP, Himachal Pradesh; ITS1, internal transcribed spacer 1; ND, not determined; VL, visceral leishmaniasis; WHO, World Health Organization. †ITS sequences strain type according to Kuhls et al. (*13*). ‡These species represent 10/44 samples used in polymorphic microsatellite analysis.

**Figure 2:**
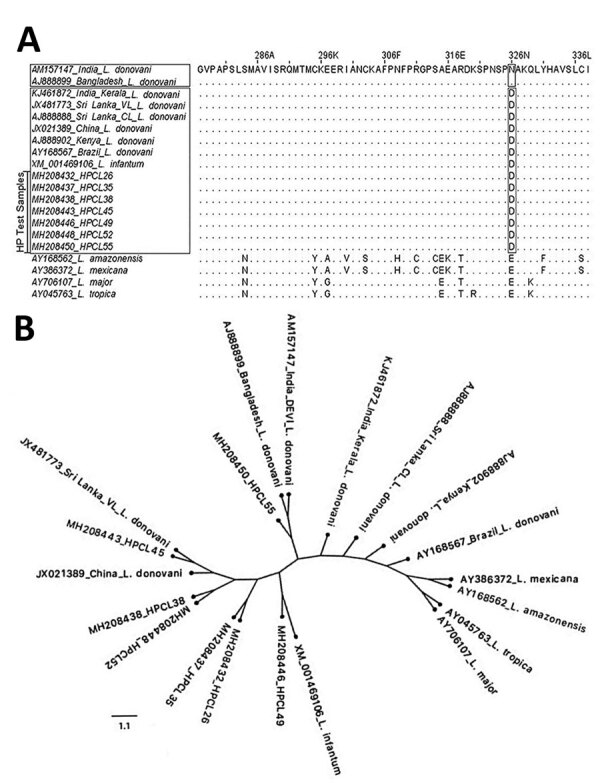
6PGDH-based molecular analysis of clinical isolates from cutaneous leishmaniasis (CL) patients, Himachal Pradesh, India, 2014–2018. A) Sequence alignment of partial 6PGDH amino acid of CL isolates exhibit replacement of asparagine (N) with aspartic acid (D) at position 326 analogous to visceral leishmaniasis–causing and CL-causing isolates from Sri Lanka. B) Phylogenetic tree for 6PGDH sequences from CL test isolates (designated as HPCL, numbered in order of their collection) and standard *Leishmania* strains. Tree constructed by using maximum-likelihood method with 5,000 bootstraps in the *dnam*l program of PHYLIP package (http://evolution.genetics.washington.edu/phylip/doc/main.html). GenBank accession numbers are indicated. Scale bar indicates the amino acid substitution per site. 6PGDH, 6-phosphogluconate dehydrogenase gene; HP, Himachal Pradesh.

Sequences of the 6-phosphogluconate dehydrogenase gene (6PGDH) exhibit a high degree of polymorphism and have been used to identify *Leishmania* species and differentiate region-specific zymodemes (*14*). We performed multiple sequence alignment of the representative partial 6PGDH amino acid sequences from Himachal Pradesh isolates by using the homologous 6PGDH protein sequences of the reference *Leishmania* isolates to determine their genetic and geographic relatedness (Table 2; Figure 2, panel A; Appendix Table 4, Figure 5). Himachal Pradesh isolates exhibited a 6PGDH sequence specific to Mon-37 and different from Mon-2 (having aspartic acid in place of asparagine) at position 326 (Figure 2, panel A). Thus, CL-causing *L. donovani* from Himachal Pradesh were distinct from the most common VL-causing India Mon-2 *L. donovani* and the Bangladesh *L. donovani* isolate, whereas they were similar to the CL-causing *L. donovani* isolate from Kerala and CL- and VL-causing Mon-37 isolates from Sri Lanka and the isolates from Kenya, Brazil, and China. 

**Table 2:** Standard *Leishmania* strains used in partial 6PGDH amino acid–based phylogenetic analysis of cutaneous leishmaniasis isolates, Himachal Pradesh, India, 2014–2018*

Species (place of origin)	WHO code	Zymodeme	GenBank accession no.	Pathology
WHO standards				
*L. donovani* (India)	MHOM/IN/0000/DEVI	MON-2	AM157147	VL
*L. major* (Turkmenistan)	MHOM/TM/1973/5ASKH	ND	AY706107	CL
* L. infantum*	ND	ND	XM_001469106	ND
* L. mexicana*	MHOM/BZ/82/BEL21	ND	AY386372	CL
* L. tropica*	ND	ND	AY045763	CL
* L. amazonensis*	ND	ND	AY168562	CL
Regional standards				
*L. donovani* (China)	MHOM/CN/90/9044	ND	JX021389	VL
*L. donovani* (Kenya)	IMAR/KE/1962/LRC–L57	MON-37	AJ888902	ND
*L. donovani* (Sri Lanka)	MHOM/LK/2010/OVN3	MON-37	JX481773	VL
*L. donovani* (Sri Lanka)	MHOM/LK/2002/L59	MON-37	AJ888888	CL
*L. donovani* (Bangladesh)	MHOM/BD/1997/BG1	ND	AJ888899	VL
*L. donovani* (Brazil)	ND	ND	AY168567	ND
*L. donovani* (Kerala, India)	ND	ND	KJ461872	CL

*6PGDH, 6-phosphogluconate dehydrogenase gene; CL, cutaneous leishmaniasis; ND, not determined; VL, visceral leishmaniasis; WHO, World Health Organization.

Phylogenetic analysis of 6PGDH amino acid sequences of CL isolates grouped them into a heterogeneous cluster; variants were closer to a viserotropic *L. donovani* isolate from Sri Lanka and distinct from the VL-causing *L. donovani* isolates from India and Bangladesh and CL-causing isolates from Kerala and Sri Lanka (Figure 2, panel B). However, the HPCL55 isolate (GenBank accession no. MH208450) grouped differently. The HPCL49 isolate (GenBank accession no. MH208446) showed relatedness to the standard *L. infantum* strain, although ITS1 analysis using BLAST and microsatellite repeat sequences showed regions of similarity with *L. donovani*. ITS1 and 6PGDH sequence analysis suggest that Himachal Pradesh isolates from CL patients consist of heterogenous *L. donovani* variants and possibly represent hybrid genotypes.

None of the CL patients had VL-specific symptoms or VL history. Ten of 43 patient blood samples tested positive for rK39 antibody, and 37 of 51 samples were positive for the circulating parasite DNA with *L. donovani*–specific ITS1 (Appendix Figure 6, panel A, B). The result suggests asymptomatic systemic *L. donovani* infection in a fraction of CL patients.

## Conclusions

The presence of leishmaniasis in Himachal Pradesh is not yet well known in India and globally (*15*). Our epidemiologic study shows newer CL pockets during 2014–2018; thus, the state needs to be recognized as leishmaniasis-endemic by public health authorities (Appendix Figure 1). We conclude that CL cases in Himachal Pradesh are caused by *L. donovani* variants distinct from the viscerotropic *L. donovani* strain from northeast India. The CL isolates in Himachal Pradesh exhibit considerable heterogeneity and indicate the possible existence of genetic hybrids. The scenario appears somewhat similar to Sri Lanka and Kerala, where *L. donovani* parasites cause cutaneous disease, albeit with differences in the region-specific *L. donovani* variants. In lieu of the coexistence of VL and CL in Himachal Pradesh, parasite isolates from VL patients also need to be characterized. To understand the biology of atypical *L. donovani* variants with cutaneous manifestations and to genetically differentiate the dermotropic versus viscerotropic potential of *L. donovani* variants, comparison of CL- and VL-causing isolates in Himachal Pradesh using whole-genome sequence analysis is required.

*L. donovani* parasites in the blood of some CL patients represent human reservoirs similar to asymptomatic VL carriers, and the parasite variants have the potential to cause full-blown VL manifestations. An elaborate surveillance program dedicated to the Himachal Pradesh region is urgently required for better diagnosis, treatment, prediction of parasite variants in different afflicted pockets, and prevention of transmission of the disease to other regions.

Appendix Additional information about *Leishmania donovani* infection with atypical cutaneous manifestations, Himachal Pradesh, India, 2014–2018.
