# Supplementary material for: Leishmania donovani Infection with Atypical Cutaneous Manifestations, Himachal Pradesh, India, 2014–2018
Source: Emerg Infect Dis. 2020 Aug;26(8):1864–9. doi: 10.3201/eid2608.191761 (PMC7392404; doi:10.3201/eid2608.191761)
Supplement: Appendix — Additional information about Leishmania donovani infection with atypical cutaneous manifestations, Himachal Pradesh, India, 2014–2018. [file 19-1761-Techapp-s1.pdf]

Article DOI: https://doi.org/10.3201/eid2608.191761

Leishmania donovani Infection with Atypical Cutaneous Manifestations, Himachal Pradesh, India, 2014–2018

Appendix

Material and Methods

Study Design and Ethics

Lesional biopsies and blood samples were collected from the CL patients reporting at Department of Dermatology, Indira Gandhi Medical College (IGMC), Shimla and Mahatma Gandhi Medical Servies Complex (MGMSC) Khaneri in Rampur, Himachal Pradesh, India. Informed consent was obtained from all the patients in the study. The study was conducted on 60 CL patients, in the age group of 4 to 70 years at the time of diagnosis over the period from 2014 to 2018. Baseline characteristics of the patients with clinical details were compiled from the standard case report forms (Appendix Table 2). Patient history regarding the visit to endemic areas, residence, place where the disease was acquired, age with lesion duration, location and distribution was recorded on the date of sample collection. Lesional 4 mm punch biopsies were taken from the active edge of the lesions from 57 patients and processed for parasite detection, histopathological and molecular studies. Blood samples were taken to access seroprevalence of rK39 antibody. The protocol of the present study was approved by the Institutional Ethics Committee IGMC, Shimla, H.P., Approval no. HFW (MS) G-5 (Ethics)/2014–10886 and Central University of Punjab, Punjab, Approval no. CUPB/IEC/2016/034.

Clinical Diagnostics

Lesional biopsy samples were processed for parasite detection using Giemsa stained touch smears, Hematoxylin and Eosin (H & E) stained paraffin-embedded tissue sections as per standard protocol (*1*,*2*). Laboratory grown reference strains, *L. donovani* and *L. major* to be used as controls in different experiments, were grown under promastigote supporting growth conditions in RPMI with 5% FBS, Penicillin (100 units/ml) and Streptomycin (100ug/ml).

A part of the lesional biopsy from the CL cases was processed for examining CL specific histopathological changes. The samples were processed in 10% NBF, embedded in paraffin and processed to 4 to 5 µm thick tissue sections. Tissue sections were stained with H&E and examined for epidermal and dermal histopathological changes specific to cutaneous lesions (*2*).

Serum Isolation and rK39 Strip Assay

Blood samples collected from 50/60 patients were used to isolate sera. The rK39 immunochromatographic rapid diagnostic test was performed to determine the seroprevalence of rK39 antibody as per manufacturer instructions (InBios International, Inc. Seattle, WA 98104). Briefly, 20 µl of serum was loaded on to the strip followed with addition of chase buffer solution. Samples were read after 10 minutes and considered positive for the presence of anti-K39 IgG with two distinct red lines corresponding to the, test region and the control region.

Molecular Analysis

Skin lesion specimens from 57/60 patients and laboratory-grown *L. donovani* and *L. major* cultures (used as controls) were used to isolate Genomic DNA (gDNA) as described previously (*3*). gDNA from patients' blood samples was also isolated to analyze presence of circulating parasite. gDNA from the test and control samples was used to perform species-specific ribosomal internal transcribed spacer 1 region (ITS1) PCR-RFLP assay and 6-phosphogluconate dehydrogenase (6PGDH) gene amplification (Appendix Figure 4, 5). Both ITS1 and 6PGDH amplification products were sequenced for species identification and to decipher genetic relatedness of the HP isolates with other region-specific VL and CL causing *Leishmania* isolates (*4*–*8*).

ITS1 PCR-RFLP

Parasite specific ITS1PCR amplification was done for all the samples with primers LITSR (5′-CTGGATCATTTTCCGATG-3′) and L5.8S (5′-TGATACCACTTATCGCACTT-3′) as described previously (*3*,*4*). Briefly 50–100 ng of gDNA was used as template and amplified with10 pmol of each primer using Go Taq Green Master mix, 1X (Promega, Cat # M7122) in a reaction volume of 25 µl. Reaction conditions comprised an initial denaturation at 95°C for 2 min, 34 cycles of denaturation at 95°C for 20 sec, annealing at 53°C for 30 sec and extension at 72°C for 1 min with the final extension at 72°C for 6 min. The PCR product of ~ 320 bp size was

subjected to *Hae*III RFLP with overnight *Hae*III digestion at 37°C and run on 2.5% agarose gel to identify the *Leishmania* species.

6PGDH PCR

6PGDH amplification was done with primers 6PGDH-F: AATCGAGCAGCTCAAGGAAG and 6PGDH-R: GAGCTTGGCGAGAATCTGAC as described previously (*7*, *8*). 50–100 ng of gDNA was amplified with 10 pmol of each primer using Go Taq Green Master mix, 1X (Promega, Cat # M7122) in a reaction volume of 25 µl. The reaction conditions comprised denaturation at 95°C for 5 min, 30 cycles of denaturation at 95°C for 1 min, annealing at 56°C for 30 secs and extension at 72°C for 1 min with the final extension at 72°C for 10 min. The PCR product was run on 1.8% agarose gel.

Sequencing and Phylogenetic Analysis

DNA sequencing of ITS1 and 6PGDH PCR products was done for identification of *Leishmania* species/species variants in relation to CL and VL causing standard *Leishmania* isolates from different regions (Table 1, https://wwwnc.cdc.gov/EID/article/26/8/19-1761-T1.htm; Table 2, https://wwwnc.cdc.gov/EID/article/26/8/19-1761-T2.htm). ITS1 PCR products from 44 samples and 6PGDH PCR products from 28 samples were outsourced for Sanger sequencing. ITS1 sequences from 44 specimens with Accession numbers MG982941 to MG982984 and 6PGDH sequences from 28 specimens with Accession numbers MH208423 to MH208450 were deposited in Genbank (Appendix Table 4).

The homologous gene sequences for ITS1 and 6PGDH of standard WHO *Leishmania* species specific isolates and region specific *L. donovani* isolates were retrieved from the NCBI-GenBank database. ITS1 nt query sequences from 44 specimens were analyzed using BLAST and multiple alignment software, MUSCLE using default parameters (*9*). The final alignment was made using BioEdit sequence alignment editor (version 7.0.5.3). The maximum likelihood tree from the aligned sequences was obtained with 5000 bootstraps with default parameters using the *dnaml* program of the phylip package (*10*). The final tree was plotted using FigTree software (version 1.4.3). To analyze 6PGDH protein sequences, gene sequences from 28 CL samples were translated into the corresponding homologous protein sequences using translate tool at ExPASy. The representative protein sequences of the seven clusters obtained from the 28 test 6PGDH protein sequences were analyzed in relation to the homologous 6PGDH protein sequences of the

reference *Leishmania* strains using the methods explained previously. The partial 6PGDH sequence alignment was made using Jalview multiple alignment editor version 2.10.4b1 (*11*). The maximum likelihood tree was obtained with 5000 bootstraps using the *proml* program of the phylip package with default parameters (*10*).

Results Section

Disease Epidemiology

During the period from 2014–2018, an increase in CL cases in the routine skin OPD was recorded in IGMC, Shimla and MGMSC, Khaneri, Rampur. A detailed record of patients suspected with CL was taken at the time of diagnosis (Appendix Table 2). In our study on 60 CL patients, there was an almost equivalent frequency of females (51.6%) and males (48.3%) ranging in age from 4 years to 70 years. The majority of the patients belonged to the indigenous students and the farming community. All the patients had localized cutaneous skin lesions predominantly on exposed body parts with involvement of the face in majority of the cases. The time gap between the appearance of lesions and disease diagnostics on the day of sample collection, ranged from 10 days to 2.6 years with most cases reporting within 3–4 months of disease occurrence, indicating a lack of awareness among local population. Most of the patients had one or two raised and itchy lesions presented as plaques, nodules and/or papules, often ulcerated unlike those present in post kala azar dermal leishmaniasis. Around 26% of cases had mucocutaneous like lesions extending to the inner nose consistent with previous reports (*12*, *13*; Appendix Table 2).

Clinical Diagnostics

Clinical confirmation of the CL cases was performed by microscopic examination of Giemsa stained lesion touch smears, H & E stained biopsy sections for the presence of LD bodies and by parasite-specific ITS1 and 6PGDH PCR analysis (Appendix Figure 2, panels A–C; Appendix Table 2). Giemsa stained tissue smears were LD positive for 50% (23/46) of the samples processed with non-availability of the smears for 14 patients. 38% (19/50) H&E stained biopsy sections were positive for LD bodies. Only 11 patients demonstrated LD positivity in both the tissue smears and the histologic sections. Patients negative for LD bodies were, however, positive for the infection based on the PCR based parasite detection. Also, the CL

lesion-specific histopathological analysis of biopsy sections performed for 50 patients exhibited characteristic dermal and epidermal changes to variable extents (Appendix Figure 3, panels A–F; Appendix Table 3). The characteristic CL lesion-specific epidermal changes with acanthosis and papillomatosis along with varying degree of keratosis accompanied granulomatous inflammation as accessed by a trained pathologist. These findings confirmed that all the 60 CL cases were positive for *Leishmania* infection and displayed varying degrees of CL specific lesional pathologies.

References

1. Bain BJ, Bates I, Laffan MA. Dacie and Lewis practical haematology e-book. London: Elsevier Health Sciences; 2016 [cited 2020 Apr 3]. https://www.elsevier.com/books/dacie-and-lewis-practical-haematology/bain/978-0-7020-6696-2

2. Lever WF, Elder DE, Elenitsas R, Johnson BL, Murphy GF. Lever's histopathology of the skin. Philadelphia: Wolters Kluwer Health; 2009.

3. Salotra P, Sreenivas G, Pogue GP, Lee N, Nakhasi HL, Ramesh V, et al. Development of a species-specific PCR assay for detection of *Leishmania donovani* in clinical samples from patients with kala-azar and post-kala-azar dermal leishmaniasis. J Clin Microbiol. 2001;39:849–54. PubMed https://doi.org/10.1128/JCM.39.3.849-854.2001

4. el Tai NO, Osman OF, el Fari M, Presber W, Schönian G. Genetic heterogeneity of ribosomal internal transcribed spacer in clinical samples of *Leishmania donovani* spotted on filter paper as revealed by single-strand conformation polymorphisms and sequencing. Trans R Soc Trop Med Hyg. 2000;94:575–9. PubMed https://doi.org/10.1016/S0035-9203(00)90093-2

5. Dávila AM, Momen H. Internal-transcribed-spacer (ITS) sequences used to explore phylogenetic relationships within *Leishmania*. Ann Trop Med Parasitol. 2000;94:651–4. PubMed https://doi.org/10.1080/00034983.2000.11813588

6. Yangzom T, Cruz I, Bern C, Argaw D, den Boer M, Vélez ID, et al. Endemic transmission of visceral leishmaniasis in Bhutan. Am J Trop Med Hyg. 2012;87:1028–37. PubMed https://doi.org/10.4269/ajtmh.2012.12-0211

7. Siriwardana HV, Noyes HA, Beeching NJ, Chance ML, Karunaweera ND, Bates PA. *Leishmania donovani* and cutaneous leishmaniasis, Sri Lanka. Emerg Infect Dis. 2007;13:476–8. PubMed https://doi.org/10.3201/eid1303.060242

8. Ranasinghe S, Zhang W-W, Wickremasinghe R, Abeygunasekera P, Chandrasekharan V, Athauda S, et al. *Leishmania donovani* zymodeme MON-37 isolated from an autochthonous visceral leishmaniasis patient in Sri Lanka. Pathog Glob Health. 2012;106:421–4. PubMed https://doi.org/10.1179/2047773212Y.0000000054

9. Edgar RC. MUSCLE: multiple sequence alignment with high accuracy and high throughput. Nucleic Acids Res. 2004;32:1792–7. PubMed https://doi.org/10.1093/nar/gkh340

10. Felsenstein J. PHYLIP (phylogeny inference package) version 3.6. 2005 [cited 2019 Dec 11]. http://evolution.genetics.washington.edu/phylip.html

11. Clamp M, Cuff J, Searle SM, Barton GJ. The Jalview Java alignment editor. Bioinformatics. 2004;20:426–7. PubMed https://doi.org/10.1093/bioinformatics/btg430

12. Thakur L, Singh KK, Shanker V, Negi A, Jain A, Matlashewski G, et al. Atypical leishmaniasis: a global perspective with emphasis on the Indian subcontinent. PLoS Negl Trop Dis. 2018;12:e0006659. PubMed https://doi.org/10.1371/journal.pntd.0006659

13. Kumari S, Garg A. Lip leishmaniasis: a new emerging clinical form of cutaneous leishmaniasis from sub-Himalayan Region. Journal of Medical Science and Clinical Research. 2018;06:62–9.

14. Sharma NL, Mahajan VK, Negi AK. Epidemiology of a new focus of localized cutaneous leishmaniasis in Himachal Pradesh. J Commun Dis. 2005;37:275–9.

Appendix Table 1. Expansion of older and newer endemic pockets of atypical CL in Himachal Pradesh

	Blocks/Tehsils		
Districts*	Newer blocks/tehsils	Earlier reported blocks/tehsils	References
Kinnaur	Kalpa, Sangla	Nichar, Pooh	(*14*)
Shimla	Shimla, Nankhari, Kotkhai, Mashobra, Theog, Chaupal, Basantpur, Rohru	Rampur, Kumarsain	(*13*)
Kullu	Banjar	Aani, Nirmand	
Newer districts	Newer blocks/tehsils		
Solan	Kunihar		
Mandi	Karsog, Thunag, Nehri, Sarkaghat		

*For older districts, reports cover all reported through 2017.

Appendix Table 2. Baseline characteristics and clinical findings: cutaneous leishmaniasis patients from Himachal Pradesh

	No. (%) by age group, y						Parasite detection, No. (%)				
Total no. of CL cases, N = 60)	0–20	21-40	>40	Persons with occupations most affected (students and farmers)	Duration till clinical diagnosis	Patients with *MCL-like lesions	Total	Giemsa touch smears LD +ve= 23/46 (50)	H&E biopsy sections LD+ve= 19/50 (38)	ITS1 PCR +ve = 47/57 (≈82)	6PGDH PCR +ve = 33/55 (60)
Male, 29/60	10/29 (34.5)	14/29 (48.3)	5/29 (17.2)	12/20 (60)	25 d to 2.6 y	6/29 (20.7)	+ve	10/21 (47.6)	10/23 (43.47)	23/57 (40.3)	16/55 (29)
							-ve	8/21 (38.1)	13/23 (56.52)	6/57 (10.5)	11/55 (20)
							Doubtful	2/21 (9.5)			
							Not Done	8/29	6/29		
Female, 31/60	8/31 (25.8)	12/31 (38.7)	11/31 (35.5)	13/20 (65)	10 d to 1.6 y	10/31 (32.3)	+ve	13/25 (52)	9/27 (33.3)	24/57 (42.1)	17/55 (31)
							-ve	10/25 (40)	18/27 (66.6)	4/57 (7)	11/55 (20)
							Doubtful	3/25 (12)			
							Not done	6/31	4/31		

Appendix Table 3. Histopathological features of CL specific lesions in ACL patients from Himachal Pradesh

Histopathological feature	No. (%) ACL cases, N = 50
Epidermal changes	
Acanthosis	28 (56)
Papilomatosis	11 (22)
Hyperkeratosis	19 (38)
Parakeratosis	15 (30)
Spongiosis	3 (6)
Subcorneal blister	7 (14)
Dermal changes	
Granuloma	22 (44)
Histiocytes	19 (38)
Epitheloid cells	39 (78)
Plasma cells	37 (74)
Giant cell	3 (6)
Lymphocytes	23 (46)
Neutrophils	30 (60)
Microabscess formation	3 (6)
Occasional eosinophils	11 (22)
Civatte bodies	1 (2)
Fibrosis	3 (6)
Spongiosis	3 (6)
LD bodies	19 (38)

*ACL, atypical cutaneous leishmaniasis

Appendix Table 4. Accession numbers of ITS1 and 6PGDH sequences of *L. donovani* isolates from dermal lesions of cutaneous leishmaniasis patients from Himachal Pradesh*

Sample no.	ITS1 accession no.	6PGDH accession no.
HPCL1	MG982941	MH208423
HPCL4	MG982942	
HPCL6	MG982943	MH208424
HPCL7	MG982944	MH208425
HPCL8	MG982945	
HPCL10	MG982946	
HPCL11	MG982947	MH208426
HPCL12	MG982948	MH208427
HPCL13	MG982949	MH208428
HPCL15	MG982950	MH208429
HPCL17	MG982951	
HPCL18	MG982952	
HPCL19	MG982953	MH208430
HPCL20	MG982954	MH208431
HPCL22	MG982955	
HPCL24	MG982956	
HPCL26	MG982957	MH208432
HPCL27	MG982958	MH208433
HPCL28	MG982959	MH208434
HPCL29	MG982960	MH208435
HPCL30	MG982961	
HPCL31	MG982962	
HPCL32	MG982963	
HPCL33	MG982964	
HPCL34	MG982965	MH208436
HPCL35	MG982966	MH208437
HPCL36	MG982967	
HPCL37	MG982968	
HPCL38	MG982969	MH208438
HPCL39	MG982970	MH208439
HPCL41	MG982971	MH208440
HPCL42	MG982972	MH208441
HPCL43	MG982973	
HPCL44	MG982974	MH208442
HPCL45	MG982975	MH208443
HPCL46	MG982976	MH208444
HPCL47	MG982977	MH208445
HPCL49	MG982978	MH208446

Sample no.	ITS1 accession no.	6PGDH accession no.
HPCL50	MG982979	MH208447
HPCL51	MG982980	
HPCL52	MG982981	MH208448
HPCL54	MG982982	MH208449
HPCL55	MG982983	MH208450
HPCL57	MG982984	

*ITS1, internal transcribed spacer 1.

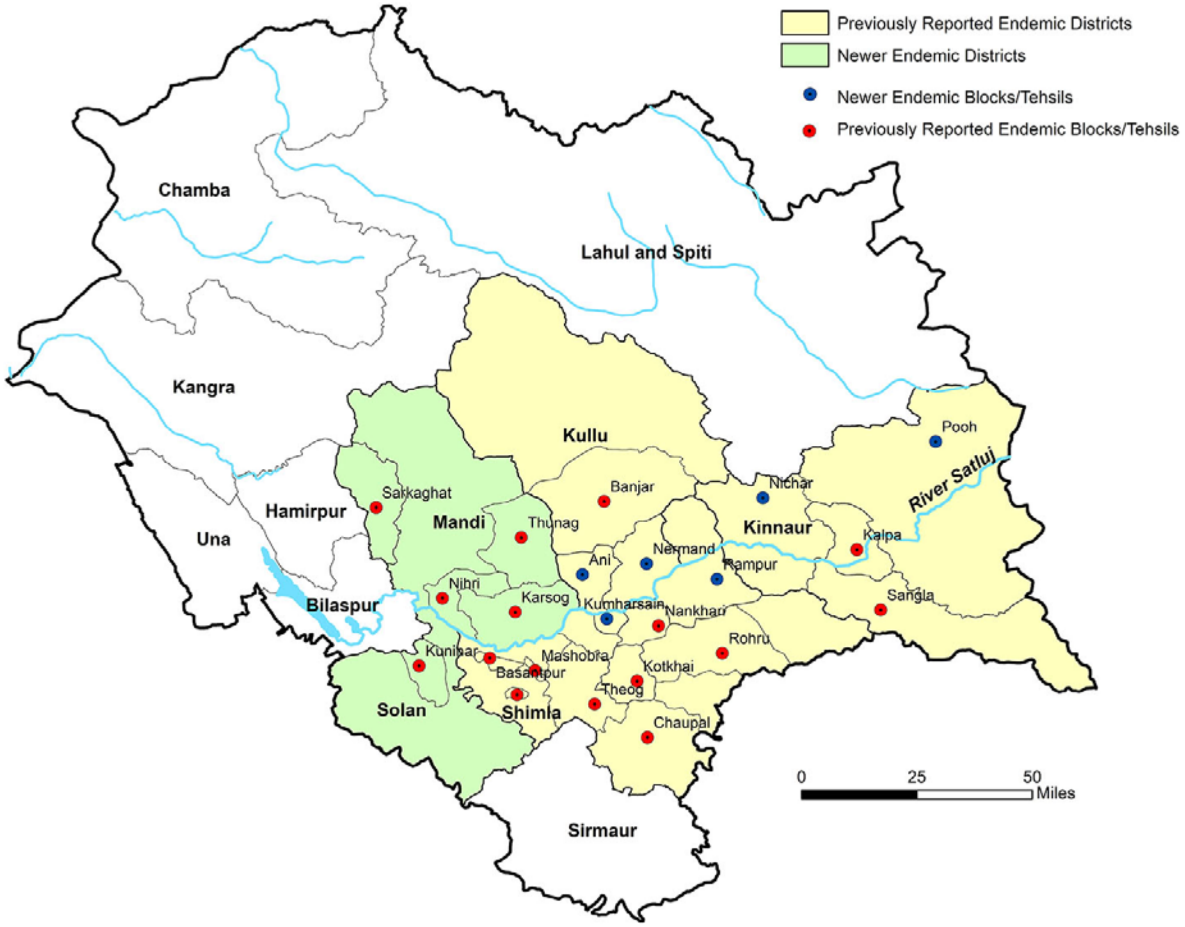

Appendix Figure 1. District map of Himachal Pradesh showing geographic distribution of atypical cutaneous leishmaniasis (ACL) cases in the newly endemic state. ACL cases from the skin OPD of reference hospitals (Indira Gandhi Medical College and Mahatma Gandhi Medical Services Complex, Shimla) and our data on patients enrolled in the study form the basis of data for disease distribution. Disease endemicity in state districts is indicated by different colors: yucca yellow shading for previously reported endemic districts and tzavorite green shading for newly emerging endemic districts. Regions with blue and red dots indicate previously reported and newly emerging endemic Blocks/Tehsils in the endemic districts of the state, respectively. The map was created using ArcGIS 10.3 software. The map showing regional distribution of disease was geo-referenced with UTM projection taking WGS84 datum. The unit of measurement for the scale bar is Kilometers.

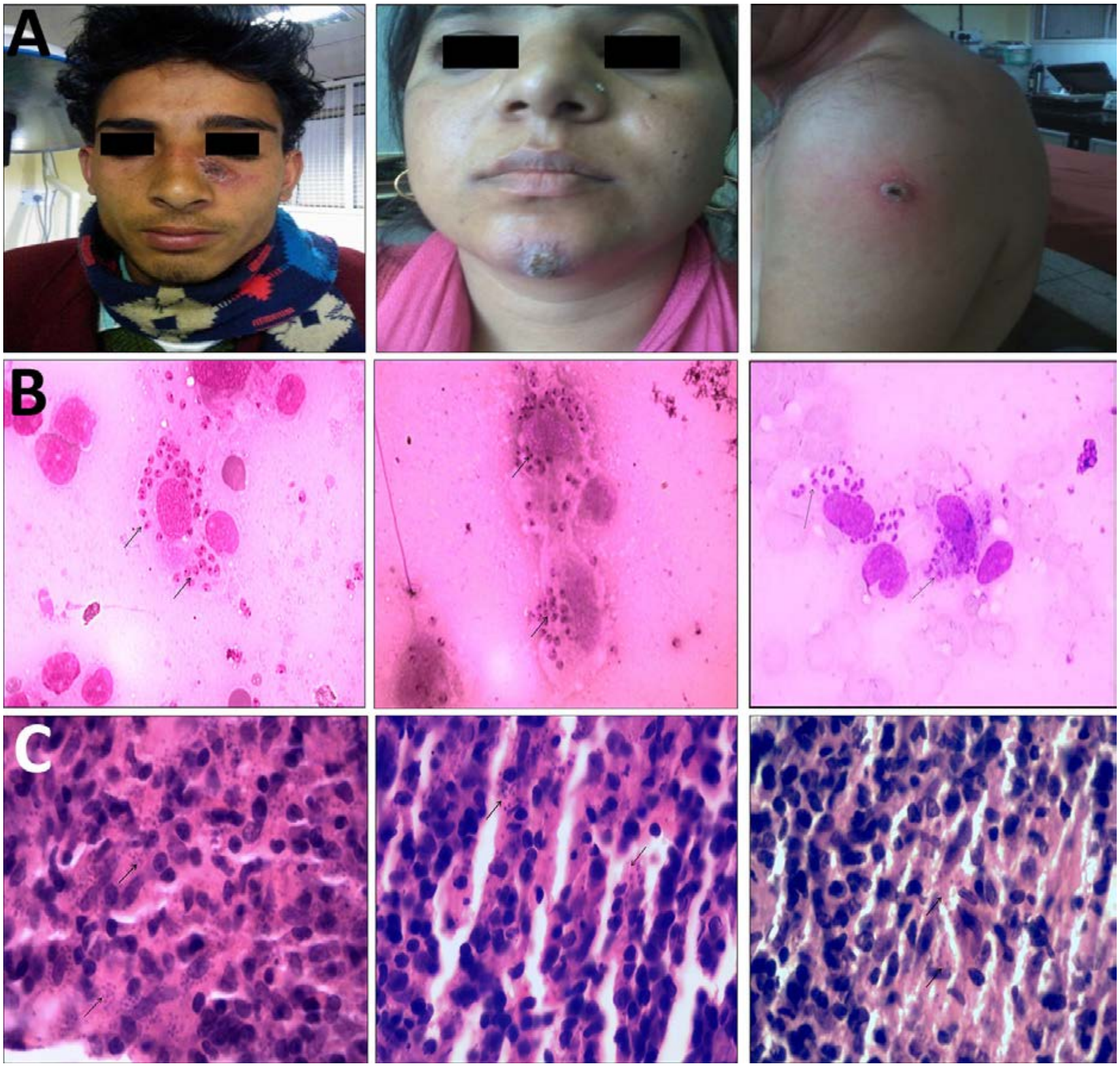

Appendix Figure 2. A) CL patients with characteristic lesions on exposed parts of the body. B) Giemsa stained tissue touch smears from patients with cutaneous leishmaniasis showing intracellular and extracellular LD bodies (arrowed). Original magnification x100. C) Hematoxylin & Eosin stained CL lesional biopsy sections showing intracellular and extracellular LD bodies (arrowed). Original magnification x100.

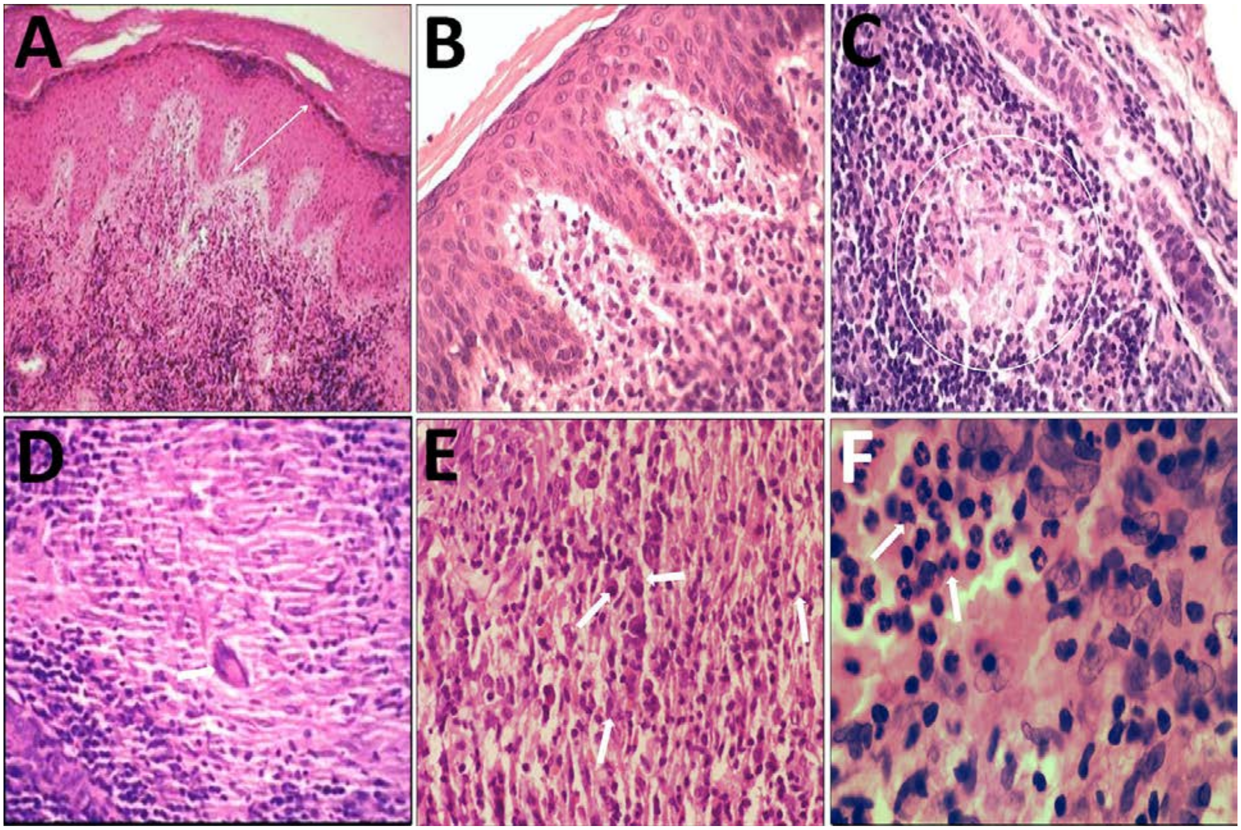

Appendix Figure 3. Histopathological characteristics of CL lesional tissue biopsies. Representative histopathological observations on Hematoxylin & Eosin stained tissue biopsy sections from CL patients. A) Mild acanthosis. Original magnification x4. B) Papilomatosis. Original magnification x10. C) Epitheloid cell granuloma (circled). Original magnification x40. D) Langhans giant cell (arrowed). Original magnification x40. E) Diffuse inflammatory cell infiltrate showing plasma cells and epitheloid cells. Original magnification x40. F) Microabscess. Original magnification x40.

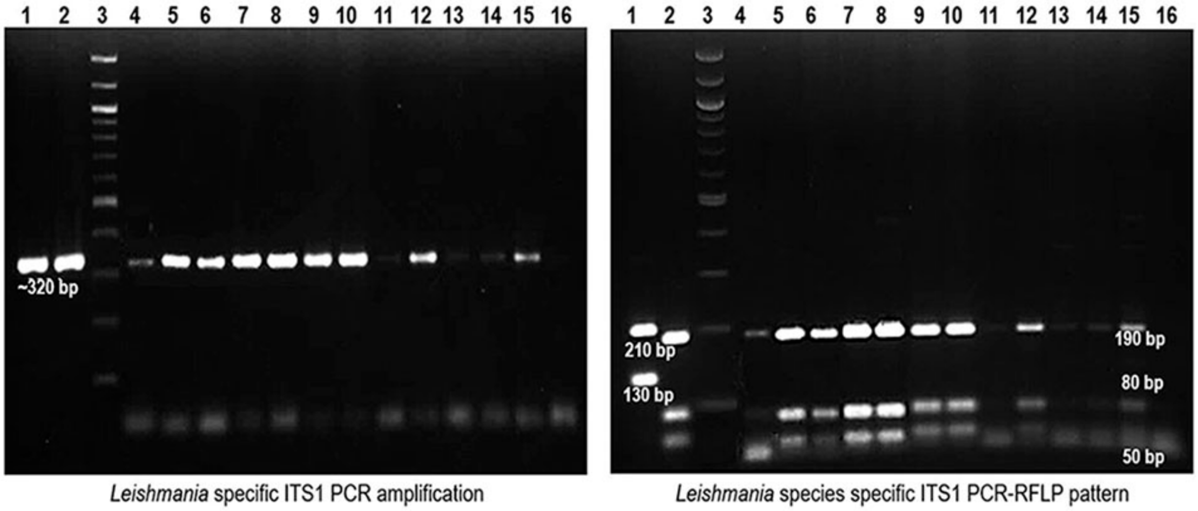

Appendix Figure 4: *Leishmania* species–specific ITS1 PCR on DNA isolated from lesional biopsy samples from cutaneous leishmaniasis (CL) patients and *Hae*III PCR–RFLP analysis of ITS1 region in test samples and standard *Leishmania* cultures used as positive controls. ITS1 PCR on cutaneous leishmaniasis patient samples. Lanes: 1, *L. major*; 2, *L. donovani*; 3, 100 bp DNA marker; 4–15, CL test samples; 16, water control. *Hae*III RFLP of ITS1 PCR amplicon for *Leishmania* species identification. Lanes: 1, *L. major*; 2, *L. donovani*; 3, 100 bp DNA ladder; 4–15, CL test samples; 16, water control. ITS1, internal transcribed spacer 1; RFLP, restriction fragment length polymorphism.

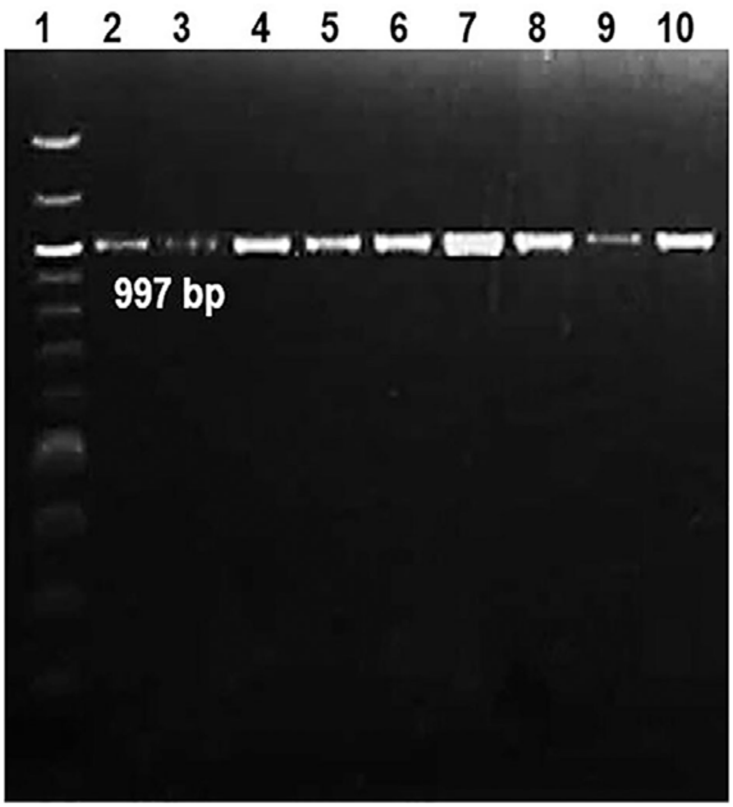

Appendix Figure 5: 6PGDH PCR on cutaneous leishmaniasis (CL) patient lesional biopsy test samples. Lanes: 1, 100 bp DNA ladder; 2, *L. donovani*; 3–10, CL patient samples from Himachal Pradesh. 6PGDH, 6-phosphogluconate dehydrogenase gene.

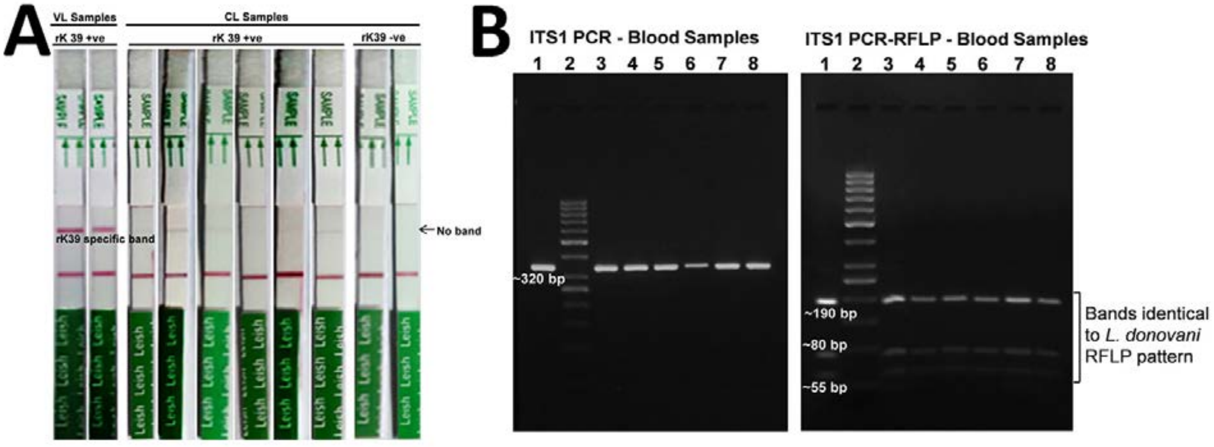

Appendix Figure 6. Systemic circulation of *L. donovani* in CL patients. A) Rapid Immuno-chromatographic rK39 dipstick test using serum samples from cutaneous leishmaniasis patients and visceral leishmaniasis patients used as positive controls for detection of circulatory *L. donovani* specific anti-rK39 antibody. The single and double band reflects negative and positive results respectively. B)

ITS1 *Leishmania* species-specific PCR on DNA from blood samples of specific ACL patients. ITS1-PCR product of ~320 bp in test samples and standard *L. donovani* positive control samples. Lanes: 1, *L. donovani* (positive control); 2, 50 bp DNA ladder; 3 to 8, CL test samples. *Hae*III PCR-RFLP analysis of ITS1. Lanes: 1, *L. donovani* (positive control); 2, 50 bp DNA ladder; 3 to 8, CL test samples.
